# The June Meetings

**Published:** 1884-07

**Authors:** 


### THE JUNE MEETINGS.

The I. H. A.—The Internationals met in Washington city, June 13th and 14th. There were present at the meeting about twenty-five members. Among these were some of the best practitioners in the homœopathic school. Many interesting papers were read, some of which we shall offer our readers later on. Altogether, the meeting was highly satisfactory, and all who were present, we believe, departed feeling refreshed and strengthened for another year's labor. Dr. R. R. Gregg was elected President. Good!

The American Institute met at Deer Park, Maryland. Attendance fuller than usual. Dr. Richard Hughes, of England, was present and the centre of attraction. Dr. J. P. Dake was appointed the American editor of the "revised" *Materia Medica* to work in unison with Dr. Hughes. No provings made with attenuations above the twelfth decimal are to be admitted into *this Materia Medica Pura.* Professor T. F. Allen was elected President for the ensuing year—excellent choice! Dr. Cowperthwaite, Vice-President—good again!

In his address next year Professor Allen can explain why his encyclopædia needs revising, and also demonstrate Dr. Dake's qualifications for the work!

